# Metabolomics Reveals Multiple Metabolic Alterations and Biomarkers of Disease Severity in Facioscapulohumeral Muscle Dystrophy

**DOI:** 10.1002/jcsm.70414

**Published:** 2026-11-06

**Authors:** Pascal Reynier, Mathieu Michel, Patrick Vandeputte, Noemi Albano, Dario Zoppi, Charlène Gillet, Sara Pini, Andi Nuredini, Martina De Maria, Odile Blanchet, Naig Gueguen, Juan Manuel Chao de la Barca, Lucia Ruggiero, Rossella Tupler, Marco Spinazzi

**Affiliations:** ^1^ Department of Biochemistry and Molecular Biology University Hospital of Angers Angers France; ^2^ Mitovasc Research Unit CNRS 6015, INSERM U‐1083, University of Angers Angers France; ^3^ Neuromuscular Reference Center, Department of Neurology University Hospital of Angers Angers France; ^4^ Department of Biomedical, Metabolic and Neural Sciences University of Modena and Reggio Emilia Modena Italy; ^5^ Department of Neuroscience, Reproductive and Odontostomatological Sciences University of Naples “Federico II” Naples Italy; ^6^ Biobank of the University Hospital of Angers Angers France

**Keywords:** ADMA, creatinine, FSHD, metabolism, metabolomics, mitochondria

## Abstract

**Background:**

Facioscapulohumeral muscular dystrophy type 1 (FSHD1) is one of the most common adult‐onset myopathies and is causally associated with a contraction of the D4Z4 macrosatellite repeat array on chromosome 4. It presents with a variable pattern of progressive muscle weakness affecting the face, shoulder girdle and lower limbs. Pathophysiological mechanisms remain incompletely understood, and no effective treatments are available. Moreover, the lack of validated biomarkers limits the assessment of disease progression and therapeutic effects in clinical trials. Metabolic dysfunction has been hypothesized as a contributing factor, yet remains poorly explored.

**Methods:**

In this multicentre, observational, cross‐sectional case–control study, we performed quantitative plasma metabolomics by mass spectrometry in 57 patients (age: 41.8 ± 11.9; females/males ratio: 1.04) with mild to moderate facioscapulohumeral muscular dystrophy (FSHD clinical score 6.4 ± 2.6) and age‐ and sex‐comparable controls (age: 41.1 ± 11.2; females/males ratio: 1.13) recruited from three tertiary neuromuscular reference centres. We restricted inclusion to patients with preserved ambulatory capacity to better capture systemic metabolic alterations not primarily driven by advanced disability. We analysed the data using an integrative framework combining univariate and multivariate analyses with five machine‐learning classifiers.

**Results:**

We identified 57 circulating metabolites discriminating patients from controls, suggesting systemic perturbations in nitric oxide, mitochondrial and one‐carbon metabolism, as well as oxidative stress, muscle mass maintenance and lipid membrane remodelling. Notably, decreased creatinine (fold change in patients vs. controls: 0.754; FDR‐corrected *p* = 0.000003) and increased asymmetric dimethylarginine (ADMA; fold change in patients vs. controls: 1.168; FDR‐corrected *p* = 0.000704) emerged as the most significantly altered metabolites, both showing strong correlations with clinical neuromuscular functional scores, including muscle strength (creatinine *ρ* = 0.62, *p* = 0.0001; ADMA *ρ* = 0.34, *p* = 0.0124), facioscapulohumeral muscular dystrophy severity score (creatinine *ρ* = 0.53, *p* = 0.0001; ADMA *ρ* = 0.32, *p* = 0.0145) and the daily activity limitation ACTIVLIM scale (creatinine *ρ* = 0.38, *p* = 0.0035; ADMA *ρ* = 0.35, *p* = 0.008). Plasma levels of GDF15, a biomarker commonly used to support the diagnosis of mitochondrial diseases, were modestly but significantly elevated in patients versus controls (542.3 pg/mL ± 277.2 vs. 444.4 pg/mL ± 164.5, *p* = 0.043). Further analysis of a predefined mitochondria‐related metabolite panel showed coordinated alterations involving mitochondrial metabolic pathways and discriminated FSHD1 patients from controls (best AUC = 0.83).

**Conclusions:**

These findings highlight systemic metabolic disturbances already detectable in mild‐to‐moderate stages of facioscapulohumeral muscular dystrophy, identifying creatinine and ADMA as promising biomarkers of disease severity, and expand current knowledge of disease pathophysiology.

**Trial Registration:** ClinicalTrials.gov identifier: NCT06086548

## 1 Introduction

Facioscapulohumeral muscular dystrophy (FSHD) is one of the most common inherited myopathies among the adult population, with a reported prevalence of about 1/12 000 [1]. It typically manifests in young adults with facial, shoulder girdle and lower limb muscle weakness [2], and atypical phenotypes are increasingly recognized. By far the most frequent form of the disease, type 1 (FSHD1), is associated with a shortening of the D4Z4 macrosatellite repeat sequence in the subtelomeric region of chromosome 4, at 4q35. However, more than 2% of the general population carries shortened D4Z4 repeats and permissive chromosome 4 haplotypes without any manifestation of this disease [3], suggesting a complex multifactorial pathogenesis. One of the most intensively studied mechanisms involves the genetic derepression of a normally silent transcription factor localized close to the D4Z4 locus, *DUX4*, inducing myogenesis inhibition and apoptosis [4]. Nevertheless, expression of *DUX4* is low and sporadic in muscle biopsies from FSHD patients [5] and is often undetectable even in affected muscle [6]. Several downstream molecular alterations have been described in FSHD, including dysregulation of *PAX7* [5] and lncRNA‐mediated effects on protein synthesis [7]. There is currently no treatment available for this disease, due to incomplete understanding of molecular pathogenesis and lack of reliable and readily accessible biomarkers to accelerate early phases of drug development [6]. Downstream pathogenic mechanisms likely participate in disease expressivity and progression but have so far been scarcely explored. Recent top‐down RNA sequencing approaches have suggested that oxidative stress and mitochondrial dysfunction are involved in FSHD1 [8]. Skeletal muscle is the most abundant tissue in the human body and actively secretes many molecules with both autocrine and endocrine effects, important for both skeletal muscle and whole‐body homeostasis. In this study, we explored the possibility that secretome and metabolic alterations may be important to understand potential actionable downstream mechanisms and to identify non‐invasive biomarkers. To investigate this hypothesis, we performed high‐throughput plasma metabolomics analysis in a deeply phenotyped cohort of patients who were mildly to moderately affected by FSHD1 and a carefully selected healthy control cohort with similar age and sex distributions to determine whether significant metabolic alterations are detectable in the early stages of the disease. By applying mass spectrometry, we generated high‐throughput metabolic profiling that we analysed by advanced computational methods, including statistical modelling and machine‐learning approaches. We complemented this unbiased approach with the analysis of the mitokine GDF15, a hormone belonging to the transforming growth factor beta superfamily released in the circulation in response to mitochondrial dysfunction. Our results reveal extensive metabolic changes and two candidate biomarkers correlating with clinical severity in FSHD1 patients.

## 2 Materials and Methods

### 2.1 Study Population

We included 57 patients, 28 males and 29 females affected by FSHD1 during the first and second quarters of 2024 (see Table 1). These were outpatients addressed for a routine examination at three tertiary neuromuscular diseases reference centres: the University Hospital of Angers, France; the Università degli Studi di Modena e Reggio Emilia, Modena, Italy; and the Università degli Studi di Napoli Federico II, Napoli, Italy.

**TABLE 1:** Demographics and disease features of study participants.

Feature	FSHD	Controls	*p*
Number	57	63	
Age, years (mean ± SD)	41.8 ± 11.9	41.1 ± 11.2	0.7407
Sex ratio (F/M)	1.04	1.13	0.8564
Body mass index, kg/m^2^ (mean ± SD)	24.8 ± 5.6	23.9 ± 3.4	0.2624
**FSHD patients' characteristics**			
Age at onset, years (mean ± SD)	21.3 ± 11.3		
Disease duration, years (mean ± SD)	20.2 ± 11.6		
Number of D4Z4 repeated units (mean ± SD)	5.6 ± 1.9		
Creatine kinase, UI/L (mean ± SD)^a^	316.7 ± 205	135 ± 90	8.18 × 10^−8^
CCEF clinical category^b^ A1 (*n*, %)	12 (21.1)		
CCEF clinical category A2 (*n*, %)	26 (45.6)		
CCEF clinical category A3 (*n*, %)	18 (31.6)		
CCEF clinical category D1 (*n*, %)	1 (1.7)		
Clinical FSHD score^c^ (mean ± SD)	6.4 ± 2.6		
Axial MRC muscles force/20 (mean ± SD)	17.1 ± 3.4		
Scapular girdle MRC muscles force/30 (mean ± SD)	24.4 ± 5.0		
Upper limb MRC muscles force/60 (mean ± SD)	56.7 ± 5.6		
Pelvic girdle MRC muscles force/40 (mean ± SD)	35.6 ± 6.3		
Lower limb MRC muscles force/40 (mean ± SD)	34.7 ± 4.9		
Total MRC muscles force score/190 (mean ± SD)	161.6 ± 20.6		
10‐m walking test, seconds (mean ± SD)	8.7 ± 4.3		
ACTIVLIM^d^ (mean ± SD)	28.4 ± 6.0		

*Note:* All demographic, anthropometric, laboratory and functional data have been collected at the time of sample collection. For categorical variables, statistical significance of proportions comparison was calculated with the exact Fisher test. For continuous variables, differences between the two groups were analysed with either a Student *t*‐test with Welch correction for normally distributed variables or a Mann–Whitney test for non‐normally distributed variables. Significance was considered for *p*‐values < 0.05.

Abbreviations: CCEF: Comprehensive Clinical Evaluation Form; FSHD: facioscapulohumeral muscular dystrophy; MRC: Medical Research Council.

^a^ CK data were available for only 33 patients and 34 controls because of limited plasma availability.

^b^ The CCEF FSHD clinical categories define different FSHD phenotypes according to the distribution of the muscle involvement and the presence or absence of atypical signs. Categories A1 to A3 define typical FSHD manifestations with different severity of facial involvement (A1: severe facial involvement with inability to close eyes and protrude lips, A2: facial involvement with upper and lower facial weakness, A3: facial involvement with upper or lower facial weakness); D1 includes one atypical manifestation.

^c^ The clinical FSHD score is a validated score for disease severity assessment in FSHD, ranging from 0 (asymptomatic) to 15 (end‐stage disease).

^d^ ACTIVLIM is a patient‐reported scale of activity limitation validated in neuromuscular diseases. It encompasses 18 items, each one being rated between 0 (impossible) and 2 (easy), ranging from 0 (patient without any autonomy in daily activities) to 36 (patient without any functional impairment in daily activities).

Inclusion criteria were the following: (1) age between 18 and 60 years; (2) molecular diagnosis of FSHD1 (number of D4Z4 repeats < 11 repeated units); (3) typical clinical features of FSHD with at least mild involvement of facial, scapular and/or pelvic girdle involvement (Medical Research Council [MRC] rating of 4/5 or below in at least one muscle of scapular and/or pelvic girdle); (4) a mild to moderate disease severity assessed by an FSHD score comprised between 4 and 10; and (5) preserved ability to ambulate at the time of the selection (use of a cane was allowed).

A comparable number of healthy controls with similar sex and age (±5 years), that is, 34 females and 30 males, were recruited during the same period. Participants were requested to be fasting at the time of blood sampling (at least 8 h).

Exclusion criteria for both patients and healthy controls were the presence of severe systemic diseases unrelated to FSHD, alcohol or toxic substance abuse, uncontrolled metabolic disease such as diabetes or hypothyroidism, or pregnancy or breastfeeding.

### 2.2 Neuromuscular Assessment

Neuromuscular assessment was performed by a neurologist specialized in neuromuscular diseases. Motor function assessment included quantification of muscle strength by MRC evaluation, 10‐m walking test, measure of daily life activity limitation by ACTIVLIM [9] and the Comprehensive Clinical Evaluation Form (CCEF) assessment of FSHD [10]. The latter allows a standardized clinical evaluation of patients affected by FSHD by classifying patients into distinct categories according to their clinical phenotype. Moreover, it provides the FSHD score, a validated scale of disease severity [10]. MRC sum score was calculated as the sum of individual MRC scores from 21 muscles (adapted from Kleyweg et al. [11]: four axial muscles, three scapular girdle muscles, six upper limb muscles, four pelvic girdle muscles and four lower limb muscles). The strength of limb muscles was assessed on both sides.

### 2.3 Blood Sample Collection

Peripheral blood samples were collected from all participants into sodium heparin tubes following an overnight fast of at least 8 h. Immediately after collection, the samples were transported on ice to the respective local laboratories, where they were processed within a short timeframe. Upon arrival, each sample was centrifuged at +4°C for 10 min at 2300 *g*. The resulting plasma was carefully aliquoted and stored at −80°C until further transport or analysis. Samples collected in Modena and Naples were subsequently shipped on dry ice to the University Hospital of Angers, France. There, they were consolidated with the locally collected samples from Angers and stored within the Biobank of the Centre de Ressources Biologiques (CRB) at the University Hospital of Angers, referenced under the Biobank Identifier BB‐0033‐00038. Haemolysed samples were excluded.

### 2.4 Metabolomic Profiling

Targeted quantitative metabolomics was performed using the MxP Quant 500 XL kit (Biocrates Life Sciences AG), in conjunction with a QTRAP 5500 mass spectrometer (SCIEX) and an Agilent 1260 HPLC system. This platform enables the simultaneous quantification of 1019 metabolites, including 107 polar compounds and 912 lipids. Additionally, 474 predefined metabolite sums and ratios, indicators of functional metabolic processes, were calculated. The kit was validated for use with the SCIEX 5500 series, ensuring reliable quantification in human heparin plasma. Sample preparation followed the manufacturer's instructions: thawed plasma was vortexed and centrifuged at +4°C for 5 min at 2800 *g*. Extraction and derivatization steps were included for amino acids and biogenic amines requiring chemical modification.

During the analytical phase, the samples were randomly distributed in the microplates of the kit to avoid a carryover effect. The analytical workflow combined flow injection analysis tandem mass spectrometry (FIA–MS/MS) for non‐polar metabolites and liquid chromatography tandem mass spectrometry (LC–MS/MS) for polar compounds. FIA–MS/MS involved injecting 20 μL of sample into a methanolic mobile phase with a proprietary additive, with flow rates ramping from 30 to 200 μL/min over a 3‐min run. LC–MS/MS used a dedicated column and precolumn at 50°C, with water and acetonitrile (both containing 0.2% formic acid) as mobile phases. In positive ion mode, the gradient transitioned from 100% aqueous to 100% acetonitrile over 5.5 min, held for 2 min and then returned to initial conditions. In negative ion mode, the acetonitrile phase reached 100% in 4.25 min and returned after 3 min. LC–MS/MS runs lasted 11 min per sample, with a 5‐μL injection volume and flow rates between 500 and 800 μL/min depending on polarity.

Mass spectrometry parameters (Q1/Q3 masses, declustering potential, collision energy and cell exit potential) were provided by the kit and optimized per compound. Data acquisition was performed using Analyst software (SCIEX), and data processing, including peak detection, identification and quantification, was carried out using MetIDQ software (Biocrates), which relies on specific MRM transitions for compound identification. Instrument performance was verified using test samples included in the kit.

Quantification was based on calibration standards and internal quality controls. LC–MS/MS used a seven‐point calibration curve with stable isotope‐labelled standards, whereas FIA–MS/MS applied a single‐point calibration. Analytical performance was assessed using lyophilized human plasma controls at low, medium and high concentrations, with one level injected in triplicate. Metabolite sums and ratios were calculated using MetIDQ software according to predefined formulas suggested by Biocrates.

### 2.5 GDF15 Measurements

Plasma GDF15 concentrations were measured using an ELISA kit (Human GDF‐15 ELISA Kit, R&D Systems), following the manufacturer's instructions, on a Clariostar microplate reader (BMG Labtech). Internal quality controls (Quantikine Immunoassay Control Group 4, R&D Systems) were included in each plate. IQCs and samples were measured in duplicate. Sample concentrations above the upper limit of the assay were controlled at a 1/2 dilution.

### 2.6 Data Processing and Statistical Analysis

Raw mass spectrometry data were processed using MetIDQ, which automates peak detection, compound identification and quantification based on predefined calibration curves. Only metabolites meeting accuracy and precision criteria (coefficient of variation < 20%) were included in statistical analyses. Metabolites with > 20% missing values (due to values below the lower limit of quantification [LOQ]) were excluded. Remaining missing values were imputed using the K‐nearest neighbours' algorithm. The resulting data matrix underwent preprocessing steps, including mean centring, unit variance scaling and logarithmic transformation.

Univariate comparisons between groups were performed using the non‐parametric Mann–Whitney *U* test. Multiple testing correction was applied using the Benjamini–Hochberg method, with a corrected *p*‐value < 0.05 considered statistically significant. However, metabolites showing the strongest significance (*p* < 0.01) but not passing false discovery rate (FDR) correction were retained to enrich biological interpretation and facilitate cross‐method comparisons.

Multivariate and machine learning methods and metabolic pathway analysis are detailed in the Supporting Information.

## 3 Results

### 3.1 Demographic Features of the Participants and Phenotypic Characterization of the FSHD Cohort

To discriminate early metabolic changes in FSHD1, we recruited 57 mild to moderately affected patients and 63 healthy controls comparable for age and sex (Table 1). One sample had been excluded since the plasma was haemolysed. Demographic and anthropometric data were not significantly different between the two groups. Age at onset, number of D4Z4 repeats, slightly elevated CK levels and clinical categories (CCEF) were consistent with typical FSHD1 cases (Table 1). Only one patient was classified in the D1 category, who presented typical FSHD manifestations and impaired shoulder abduction without winging scapula in the context of severe dorsal kyphosis. FSHD clinical severity score, muscle strength assessment, 10‐m walk test and ACTIVLIM, a scale for the assessment of limitation of daily activities in neuromuscular diseases, were consistent with mild to moderate FSHD severity (Table 1).

### 3.2 Metabolomic Analyses

Out of the 1019 metabolites analysed, 678 were considered reliably quantified based on the criteria defined in Section 2. Unsupervised principal component analysis (PCA) based on the three centres participating in the study did not reveal any clustering, indicating good standardization of our sampling procedures (Figure S1). PCA based on case and control status did not show any spontaneous clustering between groups and did identify one outlier among the cases, which was excluded from subsequent analyses (Figure S2).

Univariate analyses, considering only metabolite concentration changes greater than 10% and with a *p*‐value < 0.01, identified 21 metabolites with concentration variation (Figure 1), of which five remained significant after FDR correction (Table 2). The most notable changes included a 25% decrease in creatinine (FDR‐corrected *p* = 2.97e‐06), a 17% increase in asymmetric dimethylarginine (ADMA, FDR‐corrected *p* = 7.04e‐04), a 9% decrease in histidine (FDR‐corrected *p* = 0.003), a 12% decrease in asparagine (FDR‐corrected *p* = 0.029) and a 32% increase in lactate (FDR‐corrected *p* = 0.046).

**FIGURE 1:**
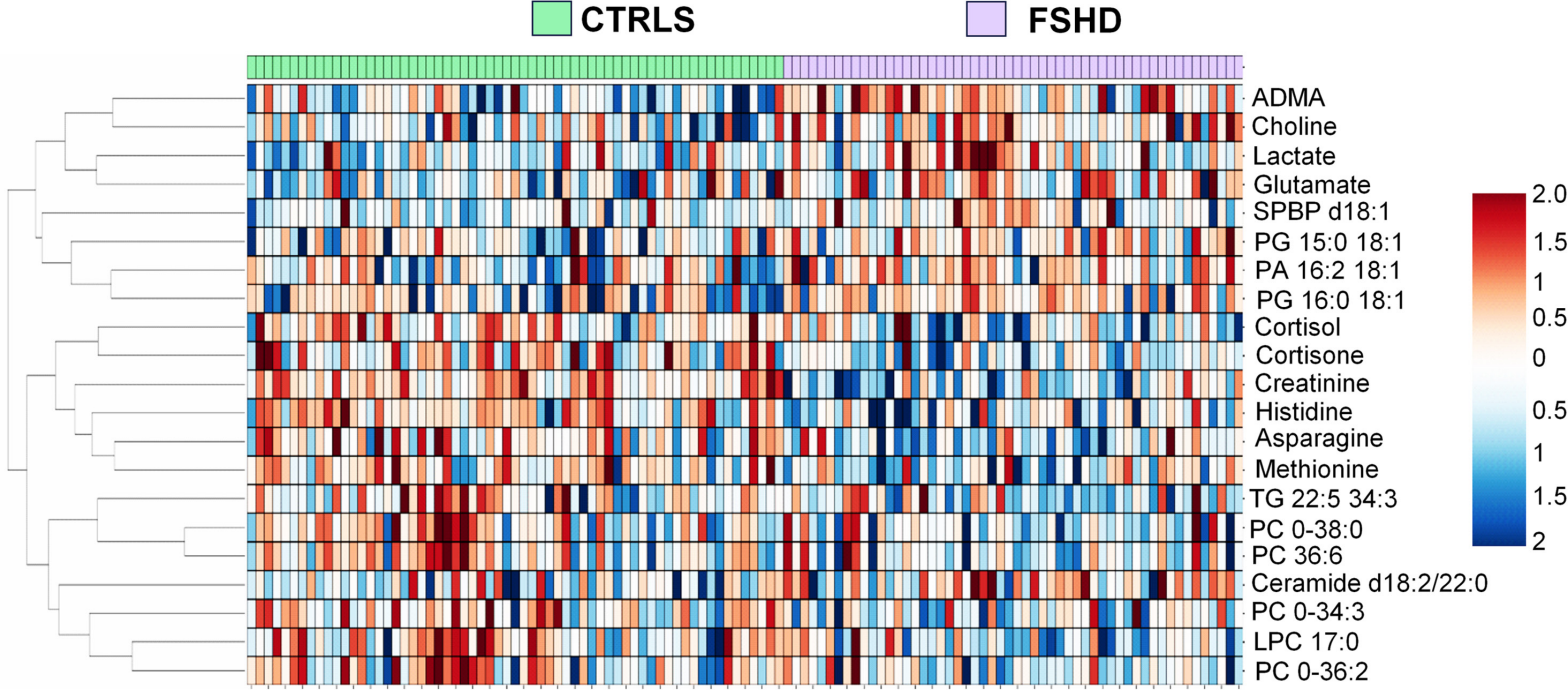
Heatmap of the 21 metabolites showing concentration differences between patients and healthy controls. In the heatmap, each column represents an individual patient or healthy control, and each row corresponds to a metabolite. In the top row, healthy controls are shown in green and FSHD patients in purple. The colour scale indicates *z*‐scores, with red representing higher‐than‐average metabolite concentrations and blue representing lower‐than‐average concentrations. ADMA: asymmetric dimethylarginine; LPC: lysophosphatidylcholine; PA: phosphatidic acid; PC: phosphatidylcholine; PG: phosphatidylglycerol; SPBP: sphingosine phosphate; TG: triglycerides.

**TABLE 2:** Univariate analysis of the variation of metabolite concentrations between FSHD patients and controls.

Metabolite	Fold change	*p*	FDR *p*
Creatinine	0.754	4.38 × 10^−9^	2.97 × 10^−6^
ADMA	1.168	2.08 × 10^−6^	7.04 × 10^−4^
Histidine	0.906	1.53 × 10^−5^	0.003
Asparagine	0.881	1.71 × 10^−4^	0.029
Lactate	1.322	3.39 × 10^−4^	0.046
Choline	1.157	5.49 × 10^−4^	0.062
Cortisone	0.879	8.09 × 10^−4^	0.069
Cortisol	0.805	8.69 × 10^−4^	0.069
LPC 17:0	0.831	9.21 × 10^−4^	0.069
Methionine	0.894	0.001	0.088
Glutamate	1.313	0.003	0.170
PC O‐34:3	0.860	0.004	0.211
SPBP d18:1	1.911	0.004	0.223
TG 22:5_34:3	0.821	0.005	0.234
PA 16:2_18:1	1.204	0.005	0.238
Cer d18:2/22:0	1.220	0.006	0.238
PC O‐38:0	0.824	0.006	0.241
PC 36:6	0.801	0.007	0.254
PG 16:0_18:1	1.418	0.007	0.254
PC O‐36:2	0.884	0.007	0.254
PG 15:0_18:1	1.352	0.009	0.292

*Note:* The table lists the 21 metabolites identified as significantly altered using the Mann–Whitney *U* test (*p*‐value < 0.01 and fold change > 1.1). The five metabolites retaining statistical significance after FDR correction are highlighted in light grey. For each metabolite, the fold change, log_2_ fold change, raw *p*‐value and FDR‐adjusted *p*‐value (Benjamini–Hochberg correction) are reported.

Abbreviations: ADMA: asymmetric dimethylarginine; Cer: ceramides; LPC: lysophosphatidylcholine; PA: phosphatidic acid; PC: phosphatidylcholine; PG: phosphatidylglycerols; SPBP: sphingosine phosphate; TG: triglycerides.

To assess global metabolic signatures discriminating between the two groups, we applied multivariate computing models including OPLS‐DA and five machine‐learning approaches. These approaches capture covariation patterns among metabolites and identify combinations of features contributing to class separation, complementing univariate results and providing an integrated view of metabolic alterations. Supervised multivariate analysis using OPLS‐DA revealed a robust predictive model distinguishing cases from controls based on metabolic profiles (Figure 2a), with a *Q* ^2^ *Y*(cum) = 0.58. The risk of overfitting was low, as demonstrated by cross‐validation ANOVA (CV‐ANOVA, *p* = 1.605e‐18) and a permutation test with a Perm *R* ^2^ of 0.352, below the threshold of 0.4. Forty metabolites had a variable importance in projection (VIP) score > 1, contributing most to the model (Figure 2b and Table S1). This multivariate signature confirmed the discriminative power of creatinine, ADMA, histidine, asparagine and lactate and also highlighted additional changes such as decreased cortisol and increased choline, glutamate and ornithine, along with alterations in triglyceride and phospholipid levels.

**FIGURE 2:**
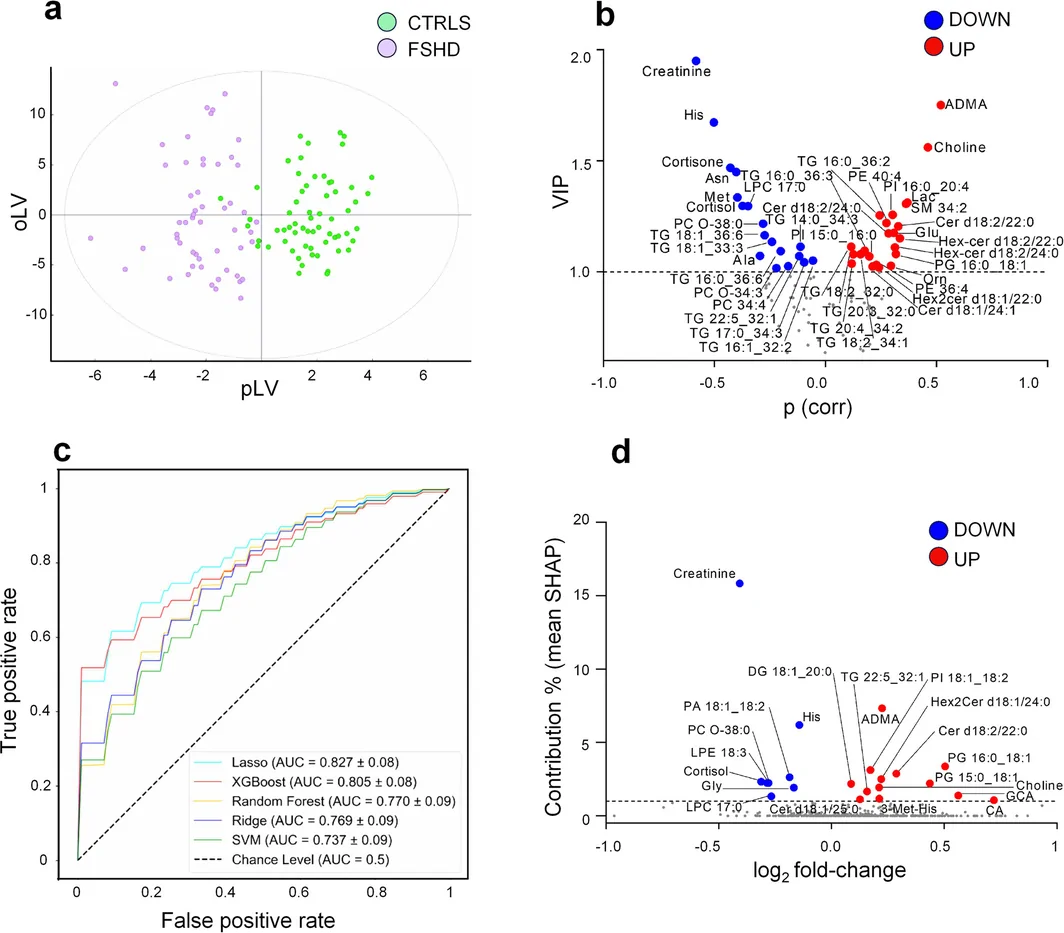
Multivariate and machine learning analysis. (a) OPLS‐DA score plot of FSHD versus control groups. OPLS‐DA score plot showing separation between the FSHD (purple) and control (green) groups along the predictive (pLV) and orthogonal (oLV) latent variables. Each point represents an individual sample, and clustering indicates distinct metabolic profiles between groups. The model explains *R* ^2^ *X* = 0.364, *R* ^2^ *Y* = 0.739, and achieves a predictive ability of *Q* ^2^ = 0.577, indicating good group discrimination despite moderate variance explained in the *X* matrix. (b) Volcano plot of the OPLS‐DA model. Metabolites with VIP > 1 are those that contribute the most to the OPLS‐DA model. The standardized loading value, *p*(corr), represents the correlation between each metabolite and the predictive component of the OPLS‐DA model. Metabolites on the left of the volcano plot with a negative *p*(corr) have decreased concentrations in FSHD compared to controls, whereas those on the right with a positive *p*(corr) have increased concentrations. VIP: variable importance in the projection. *p*(corr): correlation‐scaled loading *p*. (c) Receiver operating characteristic (ROC) curves for different machine‐learning models. ROC area under the curve (AUC) plots showing the mean AUC values achieved by all machine‐learning models tested (logistic regression with Lasso and Ridge penalization, random forest, XGBoost and SVM), with Lasso yielding the best performance with an AUC of 0.827. (d) SHAP volcano plot for the Lasso model. In this volcano plot, the *x*‐axis indicates the log_2_ fold change, whereas the *y*‐axis represents the percentage contribution to the Lasso logistic regression model, computed from SHAP values. Metabolites positioned on the left have reduced concentrations in FSHD, whereas those on the right exhibit elevated concentrations relative to controls. 3‐Met‐His: 3‐methylhistidine; ADMA: asymmetric dimethylarginine; Ala: alanine; Asn: asparagine; CA: cholic acid; Cer: ceramides; DG: diglycerides; GCA: glycocholic acid; Glu: glutamine; Gly: glycine; Hex‐Cer: hexosylceramides; His: histidine; Lac: lactate; LPC: lysophosphatidylcholine; LPE: lysophosphatidylethanolamines; Met: methionine; Orn: ornithine; PA: phosphatidic acid; PC: phosphatidylcholine; PE: phosphatidylethanolamine; PG: phosphatidylglycerol; PI: phosphatidylinositols; SM: sphingomyelins; SPBP: sphingosine phosphate; TG: triglycerides.

Machine learning analysis using five algorithms demonstrated predictive power for case/control classification based on the metabolic signature, with all models achieving an area under the curve (AUC) > 0.7. The best performance was obtained with the Lasso algorithm (Figure 2c, AUC = 0.83; Table S2). SHAP value analysis identified 21 key metabolites contributing to the Lasso model, again highlighting creatinine, ADMA, histidine, choline and cortisol, along with several lipid species (Figure 2d).

Figure 3a summarizes these findings. The Venn diagram illustrates the overlap of metabolites highlighted by the different analytical approaches, with metabolites identified by three or two methods considered the most robust. For the univariate approach, all 21 metabolites with nominal significance (*p* < 0.05) were included, whereas only five remained significant after FDR correction. Metabolites identified by a single analytical approach were retained to provide a broader overview of the metabolic signature.

**FIGURE 3:**
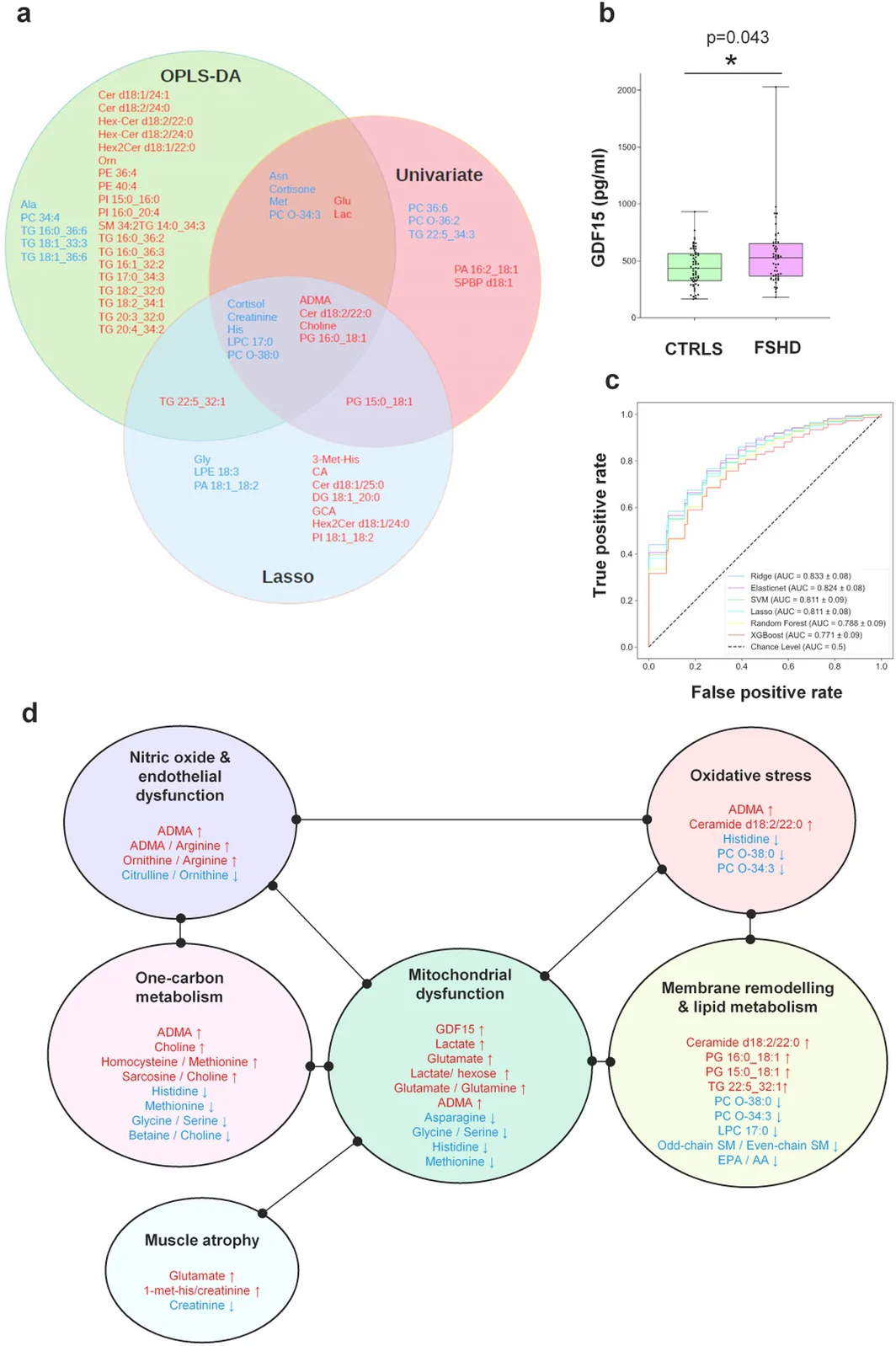
Integrative metabolomic analysis and validation of mitochondrial dysfunction by GDF15 analysis. (a) Venn diagram showing the metabolites significantly altered in FSHD patients by a combination of multivariate methods with OPLS‐DA (VIP > 1), Lasso (% mean SHAP value > 1%) and univariate analyses using the Mann–Whitney *U* test (*p*‐value < 0.01 and fold change > 1.1). Metabolites with increased concentration in FSHD are presented in red, and those with decreased concentrations are presented in blue. The most important metabolites are shown at the intersections between the circles. (b) Quantification of GDF15 plasma levels. GDF15 is significantly increased in FSHD compared to controls (542.3 ± 277.2 vs. 444.1 ± 164.4, *p*‐value = 0.0431 by the Mann–Whitney test). (c) ROC curves of supervised machine‐learning models based on a manually curated panel of mitochondria‐related metabolites. Receiver operating characteristic (ROC) curves illustrating the classification performance of six supervised machine‐learning models in discriminating FSHD patients from controls using a predefined panel of metabolites manually selected from the quantified metabolome based on published evidence of direct or indirect involvement in mitochondrial dysfunction. The Ridge regression model achieved the highest performance (AUC = 0.833 ± 0.08), followed by elastic net, support vector machine (SVM), Lasso, random forest and XGBoost. The dashed diagonal line represents the performance of a random classifier (AUC = 0.5). Model performance is reported as mean AUC ± standard deviation across repeated cross‐validation folds. (d) Functional assignment of the discriminating metabolites identified by at least two independent computational methods as illustrated in (a), GDF15 and significant metabolic ratios (Table 2) to metabolic pathways categories according to the literature. Metabolites with increased concentration in FSHD are presented in red, and those with decreased concentrations are presented in blue. The lines indicate overlapping functions in different functional assignments of one or more metabolites. 3‐Met‐His: 3‐methylhistidine; ADMA: asymmetric dimethylarginine; Ala: alanine; Asn: asparagine; CA: cholic acid; Cer: ceramides; DG: diglycerides; GCA: glycocholic acid; Glu: glutamine; Gly: glycine; Hex‐Cer: hexosylceramides; His: histidine; Lac: lactate; LPC: lysophosphatidylcholine; LPE: lysophosphatidylethanolamines; Met: methionine; Orn: ornithine; PA: phosphatidic acid; PC: phosphatidylcholine; PE: phosphatidylethanolamine; PG: phosphatidylglycerol; PI: phosphatidylinositols; SM: sphingomyelins; SPBP: sphingosine phosphate; TG: triglycerides.

An important consideration is whether the identified metabolites arise from independent biochemical mechanisms or whether they reflect coordinated perturbations within a few central metabolic pathways. Analysis of the 400 sums and ratios proposed by Biocrates identified 14 significantly altered sums or ratios in cases compared to controls after FDR correction (Table 3). Most of these were directly or indirectly related to discriminant metabolites such as ADMA, choline, ornithine, lactate and glutamate. This approach revealed disruptions in several metabolic functions, including nitric oxide biosynthesis, enzymes related to one‐carbon metabolism, muscle protein degradation, inflammation and mitochondrial function (lactate/hexose, glutamate/glutamine and glycine/serine). These latter, in association with the identification of increased lactate in patients, coherently indicate mitochondrial dysfunction in FSHD1. To obtain independent evidence supporting this hypothesis, we quantified circulating GDF15, a cytokine sensitive to cellular stress and widely employed as a biomarker of mitochondrial disorders [12], and observed a significant increase in patients compared to controls (542.3 ± 277.2 vs. 444.4 ± 164.5, *p* = 0.043; Figure 3b).

**TABLE 3:** Metabolite ratios.

Metabolite ratios	Functional significance	Fold change	*p*	FDR *p*
ADMA/arginine	Asymmetrical arginine methylation	1.279	4.46 × 10^−6^	4.23 × 10^−4^
Sarcosine/choline	Sarcosine synthesis from choline	0.776	8.97 × 10^−5^	0.004
Citrulline/ornithine	Citrulline synthesis	0.831	1.63 × 10^−4^	0.004
Lactate/hexose	LDH activity	1.332	2.15 × 10^−4^	0.004
1‐Methylhistidine/creatinine	Muscle protein degradation	1.230	3.84 × 10^−4^	0.006
Glutamate/glutamine	Glutaminase activity	1.359	4.81 × 10^−4^	0.007
Odd‐chain/even‐chain sphingomyelins	Altered fatty acid metabolism	0.901	8.37 × 10^−4^	0.010
Homoarginine/ADMA	Inhibition of NO synthesis	0.812	0.001	0.011
Glycine/serine	GLY synthesis/mitochondrial SHMT2	0.885	0.002	0.017
Homocysteine/methionine	Homocysteine synthesis	1.327	0.002	0.017
Betain/choline	Betain synthesis	0.853	0.003	0.027
Ornithine/arginine	Arginase/ornithine synthesis	1.223	0.006	0.042
Ratio of EPA (FA20:5) to AA (FA20:4)	Inflammatory marker	0.789	0.006	0.043

*Note:* The table lists the metabolite ratios identified as significantly altered using the Mann–Whitney *U* test (*p*‐value < 0.01 and fold change > 1.1) after FDR correction of the *p*‐value using Benjamini–Hochberg correction.

Abbreviations: AA: arachidonic acid; ADMA: asymmetric methylarginine; EPA: eicosapentaenoic acid; FA: fatty acid; GLY: glycine; LDH: lactate dehydrogenase; NO: nitric oxide; SHMT2: serine hydroxymethyltransferase.

To further investigate whether the observed metabolic alterations were consistent with mitochondrial dysfunction, we performed pathway enrichment analysis using a predefined literature‐derived panel of quantified metabolites previously associated with mitochondrial diseases or impairment (Table S3). This analysis identified significant enrichment of multiple pathways, notably those related to the metabolism of pyruvate, alanine, aspartate and glutamate, arginine, one‐carbon, cysteine and methionine (Figure S3). We next evaluated the ability of this mitochondria‐associated metabolite panel to discriminate FSHD patients from controls using supervised machine‐learning models. Among the six algorithms tested, ridge regression achieved the best classification performance, with a mean cross‐validated AUC of 0.833 ± 0.08, followed by elastic net, support vector machine, lasso, random forest and XGBoost (Figure 3c). These findings indicate that this subset of mitochondria‐related metabolites provides good discrimination between FSHD patients and controls. Altogether, functional categorization of the metabolic alterations identified in FSHD1 highlights perturbations in several interconnected biological processes, including mitochondrial metabolism, oxidative stress, nitric oxide biosynthesis, one‐carbon metabolism, muscle mass homeostasis and membrane remodelling (Figure 3d).

### 3.3 Correlations of Creatinine and ADMA Plasma Levels With Multiple Clinical Measures of FSHD Disease Severity

Clinically useful biomarkers should ideally reflect disease severity, allowing for objective monitoring of disease progression. There is no such biomarker yet identified for FSHD. To identify putative biomarkers of disease severity, we performed correlative analysis between the most discriminative metabolites (Figure 3a) and three independent validated and clinically relevant measures of neuromuscular function. We found that plasma creatinine and ADMA levels significantly correlated with all three functional scores (Figure 4a,b). We conclude that both creatinine and ADMA levels significantly correlate with disease severity in FSHD1.

**FIGURE 4:**
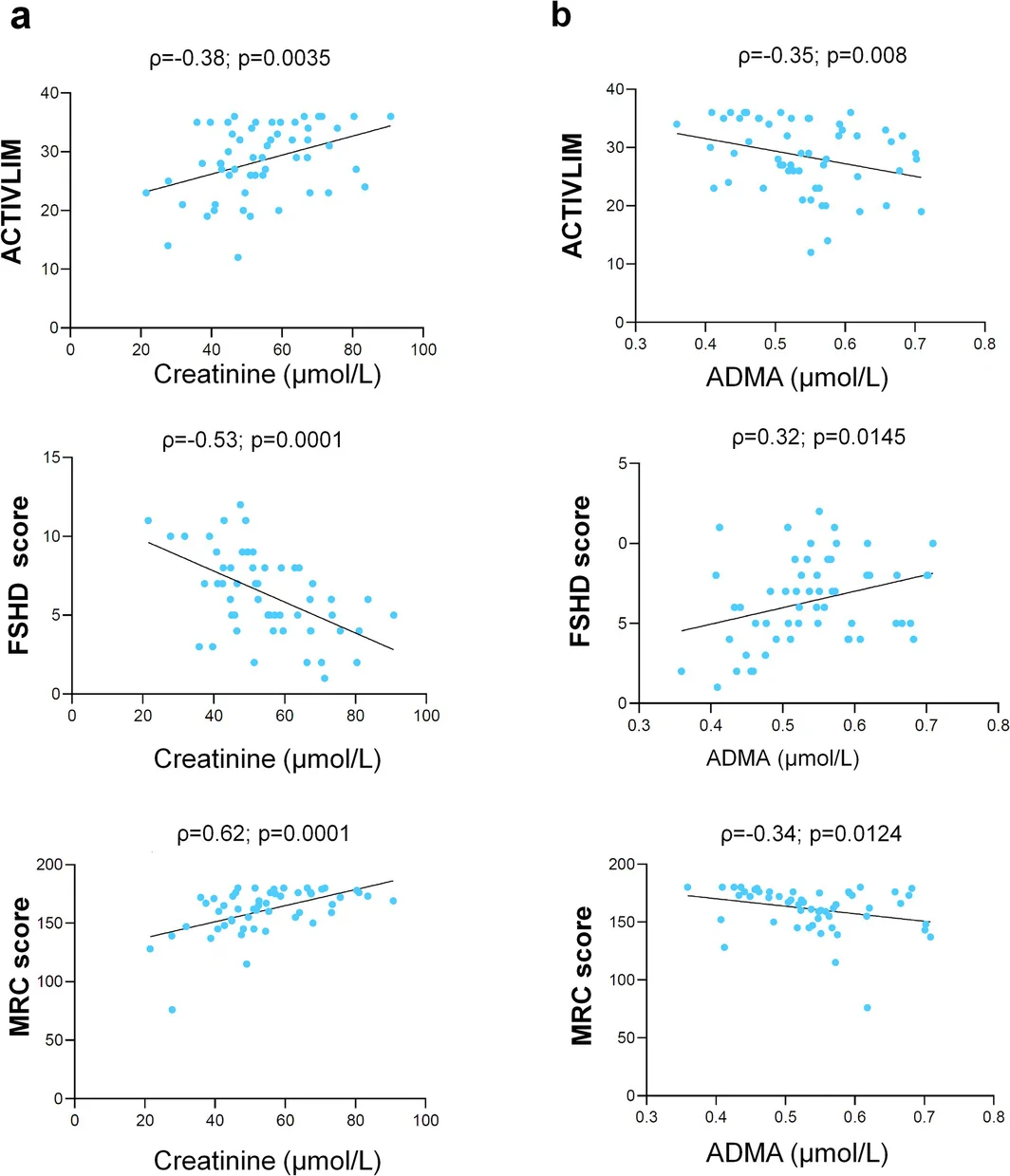
Correlations of creatinine and ADMA levels with multiple neuromuscular function scores. (a) Linear correlation of creatinine plasma levels in FSHD patients with the daily activity limitation score ACTIVLIM (top), FSHD clinical score (middle) and muscle strength assessed by the MRC sum score (bottom). Statistical significance was calculated by computing Spearman correlation coefficients, *ρ*. (b) Spearman correlation of ADMA plasma levels with the daily activity limitation score ACTIVLIM (top), FSHD clinical score (middle) and muscle strength assessed by the MRC sum score (bottom).

## 4 Discussion

Few studies have assessed metabolic function in FSHD using metabolomics [13, 14]. Two studies were performed on cohorts of different muscle diseases, which included small groups of FSHD1 patients [13, 14], and reported a limited number of significant changes but lacked detailed clinical phenotyping and correlations with disease severity. In our study, we included a large cohort of carefully phenotyped FSHD1 patients and controls with similar age and sex distribution. Importantly, we focused on moderately affected patients to capture metabolic changes reflecting pathogenic mechanisms activated in early stages of the disease rather than secondary consequences of late‐stage muscle degeneration. The multicentre nature of our study enhances the robustness and generalizability of our findings, mitigating local biases and enabling internal replication across independent cohorts. We used a quantitative analytical platform and the combination of statistics and machine learning methods to improve group separation, reduce confounding variability and uncover latent metabolic signatures, thereby facilitating robust biomarker and disease mechanism discovery. Such an approach led us to discover a broad plasma metabolomic signature in FSHD1 patients compared to healthy controls, comprising 57 significantly altered metabolites, 37 increased and 20 decreased. Among these, nine emerged as the most discriminating, consistently identified across all three analytical approaches (univariate analysis, OPLS‐DA and machine learning), and 15 were reproducibly validated by at least two methods. Furthermore, among a list of over 400 biologically relevant metabolite sums and ratios interrogating different metabolic functions, 14 were found significantly altered after FDR correction (Table 2). Most of these alterations have not been previously reported in FSHD. These metabolite changes indicate that FSHD1 is associated with systemic metabolic alterations belonging to different functional pathways including nitric oxide/endothelial metabolism, mitochondrial function, oxidative stress, one‐carbon metabolism, muscle mass maintenance and membrane remodelling. The key circulating metabolic markers identified in the study, together with their role in metabolism, muscle physiology and reported links with neuromuscular diseases, are presented in Table S4.

### 4.1 Creatinine, a Marker of Muscle Mass, Strongly Correlates With FSHD Clinical Severity

The decrease in circulating creatinine levels (−25%; FDR *p*‐value = 0.000003) in FSHD patients was the strongest discriminant metabolite of our signature. Given its large effect size, robust correlation with neuromuscular functional measures, high analytical precision and low assay costs, creatinine emerged as a particularly informative marker among the identified metabolites. Creatinine is a byproduct of creatine metabolism and is recognized as a surrogate marker of muscle mass [15]. Accordingly, reduced serum and urinary creatinine levels have been reported in several conditions associated with muscle atrophy such as Duchenne muscular dystrophy (DMD) and other neuromuscular diseases [16, 17, 18]. A recent longitudinal study involving 555 DMD patients has shown a significant correlation between plasma and urinary creatinine and motor function decline [16]. Although the underlying mechanisms differ between DMD and FSHD, both conditions share progressive muscle atrophy, which may explain the observed decrease in creatininaemia. Thus, while this association is not unexpected, to our knowledge, this is the first report documenting such a relationship in FSHD. Similarly, the more modest increases in plasma glutamate [19] and 3‐methylhistidine (3‐Met‐His) [20] are consistent with increased muscle catabolism. Future studies are required to correlate creatinine levels and muscle mass measurements. Importantly, we show that creatinine levels significantly correlate with clinically relevant neuromuscular functional parameters such as muscle strength, FSHD score [10] and ACTIVLIM [9]. These findings support the value of creatinine as a non‐invasive indicator of disease severity. While not necessarily reflecting disease‐specific molecular mechanisms, circulating creatinine may nonetheless provide clinically useful information on the global muscle status of patients. This may be particularly relevant in FSHD, where marked asymmetry, heterogeneous muscle involvement and variable progression complicate the assessment of muscle mass using limb morphometry (i.e., circumference measurements) of selected muscles. Longitudinal studies are required to determine whether creatinine levels may serve as a marker of disease progression in follow‐up evaluations and clinical trials.

### 4.2 ADMA, a Metabolite Involved in Vascular Homeostasis, Correlates With FSHD Severity

Among the most discriminant metabolites, ADMA also stood out prominently. Its plasma concentration was significantly elevated in FSHD1 patients (+17%, FDR‐corrected *p*‐value = 0.0007). The ADMA‐to‐arginine ratio, reflecting asymmetric arginine methylation, showed the strongest statistical significance among all calculated ratios and sums (+28%, FDR *p*‐value = 0.0004). To our knowledge, this is the first report of dysregulated dimethylarginines in FSHD. Moreover, we found that ADMA plasma levels significantly correlate with three independent measures of FSHD clinical severity (ACTIVLIM, muscle strength and FSHD score). ADMA is generated by proteolytic degradation of proteins containing post‐translationally methylated arginine residues, using S‐adenosyl‐methionine (SAM) as a one‐carbon donor [21]. Elevated ADMA levels are generated by oxidative stress and inflammation through parallel decreased ADMA degradation and increased production [22]. ADMA is a well‐established endogenous inhibitor of nitric oxide synthase (NOS). NO biosynthesis is critical for endothelial, vascular and muscle homeostasis. Accordingly, increased ADMA may reduce nitric oxide generation, inducing endothelial dysfunction and increased cardiovascular risk [23]. Interestingly, ADMA has recently been shown to be transported into mitochondria through SLC25A45, although its intramitochondrial role and potential effects on mitochondrial nitric oxide metabolism remain unknown [24]. Altogether, ADMA metabolism is interconnected with several cellular processes, including nitric oxide biosynthesis, one‐carbon metabolism, mitochondrial function and oxidative stress.

Elevated ADMA levels have been reported in numerous chronic disorders, particularly those associated with endothelial dysfunction, including hypertension, vascular disease, chronic kidney disease, heart failure and diabetes [21, 22]. Increased ADMA has also been observed in neurological disorders such as amyotrophic lateral sclerosis, where it correlates with disease progression [25], and in the mitochondrial disease MELAS [26]. Furthermore, higher circulating ADMA levels predict mortality in critically ill patients [27] and are associated with age‐related loss of muscle strength in older adults [28]. Interestingly, plasma ADMA concentrations were reported to be unchanged in patients with DMD despite alterations of the l‐arginine/nitric oxide pathway [29]. By contrast, increased ADMA levels have been described in the mdx mouse model, in which dystrophin deficiency disrupts the sarcolemmal localization of nNOSμ, leading to impaired NO signalling and functional muscle ischaemia [30, 31]. In our cohort, disruption of the arginine–NO pathway is further supported by significant alterations in the citrulline/ornithine and ornithine/arginine ratios, the latter reflecting arginase activity [32]. Moreover, muscle oxygenation has been shown to be reduced in patients with FSHD [8]. Taken together, these findings identify a previously unrecognized perturbation of arginine–NO metabolism in FSHD that may contribute to endothelial dysfunction and impaired skeletal muscle perfusion. The mechanisms underlying increased ADMA levels in FSHD remain unknown and warrant further investigation. These findings raise the possibility that therapeutic strategies aimed at restoring arginine–NO homeostasis may deserve evaluation in FSHD.

### 4.3 Multiple Metabolomic Changes Involve Mitochondria‐Related Pathways

Our study identified several systemic metabolic changes involving pathways related to mitochondrial metabolism. We observed a significant increase in blood lactate levels in FSHD patients, as well as an increased lactate/hexoses ratio. Elevated lactate is a well‐established diagnostic marker in mitochondrial diseases, where it reflects increased reliance on glycolysis associated with impaired oxidative phosphorylation.

Other metabolomic changes such as increased glutamate, decreased asparagine, decreased glutamate/glutamine and decreased glycine/serine ratios involve metabolic pathways closely linked to mitochondrial function [33]. The glutamate/glutamine ratio reflects glutaminase activity, which may be altered in conditions of defective mitochondrial metabolism [12]. Moreover, alterations in serine biosynthesis and one‐carbon metabolism had been observed in models of mitochondrial DNA defects [33].

We also analysed the concentration of GDF15, a cytokine widely used as a peripheral biomarker in mitochondrial disease diagnosis [12]. GDF15 was modestly but significantly increased in patients compared to controls with a similar age and sex. It should be noted, however, that GDF15 is sensitive but not specific for mitochondrial diseases and can be increased in several other conditions, including ageing and sarcopenia associated with cancer, heart and lung diseases, and myositis [34]. GDF15 is a general cellular stress‐responsive cytokine that can be induced as part of the integrated stress response and by several pathophysiological processes, including mitochondrial dysfunction, endoplasmic reticulum stress, oxidative stress, hypoxia and inflammation. These processes are themselves frequently interconnected.

Pathway analysis using a predefined literature‐derived panel of mitochondria‐related metabolites identified multiple significantly enriched pathways related to mitochondrial metabolism. Moreover, supervised machine‐learning models based on this predefined mitochondrial metabolite panel consistently discriminated FSHD patients from controls (Figure 3c).

Although the contribution of mitochondrial dysfunction in FSHD has been little explored, previous studies have shown morphological, functional and gene expression profiles consistent with mitochondrial dysfunction [8, 35, 36]. Reduced aerobic capacity has been previously reported in FSHD by exercise testing [37]. Altogether, our findings identify a systemic metabolic signature involving several mitochondria‐related metabolites and pathways, which is compatible with, although does not formally demonstrate, the presence of mitochondrial dysfunction in FSHD. Further studies with direct mitochondrial assessment in skeletal muscle will be required to determine the extent to which the identified metabolic signature reflects mitochondrial dysfunction versus other biological processes, the precise mechanisms of mitochondrial dysfunction and its pathophysiological relevance.

### 4.4 Decreased Plasma Histidine Levels in FSHD1

Our analysis identified plasma histidine as the most significantly decreased amino acid in FSHD, confirming previous findings obtained by NMR spectroscopy in a smaller FSHD cohort [13]. Interestingly, the same group reported increased histidine concentrations in FSHD muscle tissue, suggesting altered systemic histidine homeostasis [38]. Reduced circulating histidine has also been described in inflammatory and metabolic disorders, cancer cachexia [39, 40] and mitochondrial diseases [12, 41]. Histidine contributes to cellular antioxidant defence both directly, through its imidazole ring, and indirectly as a precursor of carnosine. In addition, histidine catabolism is linked to one‐carbon metabolism and NADPH‐dependent redox homeostasis. Therefore, decreased circulating histidine is consistent with increased oxidative stress, a mechanism that has previously been implicated in FSHD pathophysiology [8, 35, 42, 43].

### 4.5 One‐Carbon Metabolism

We identified significant alterations in several metabolites involved in one‐carbon metabolism, including histidine, choline, methionine and ADMA, together with changes in metabolite ratios reflecting different steps of this pathway (glycine/serine, homocysteine/methionine, betaine/choline and sarcosine/choline). To our knowledge, these alterations have not previously been reported in FSHD. One‐carbon metabolism is closely interconnected with mitochondrial metabolism, redox homeostasis and methylation reactions [33]. However, the mechanisms responsible for these alterations and their potential functional consequences in FSHD remain to be established.

### 4.6 Limitations

The present study has several limitations. First, owing to its cross‐sectional design, it cannot establish causal or hierarchical relationships among the metabolic alterations identified. The observed metabolic changes may represent upstream pathogenic mechanisms, downstream consequences of muscle degeneration, adaptive responses or a combination thereof. Second, the absence of disease control groups precludes assessment of the specificity of the identified metabolic signature for FSHD. Finally, although our findings identify metabolic alterations involving several mitochondria‐related metabolites and pathways, they do not directly demonstrate mitochondrial dysfunction or establish to what extent mitochondrial dysfunction contributes to the identified metabolic signature. Other processes, including hypoxia, oxidative stress, inflammation or muscle degeneration, may contribute to these alterations, either independently or in conjunction with mitochondrial dysfunction. Longitudinal studies, direct functional assessment of mitochondria in skeletal muscle, mechanistic investigations and analyses in other neuromuscular disorders will be required to clarify these relationships.

### 4.7 Conclusions

This study provides a comprehensive plasma metabolomic profiling of patients with FSHD and identifies a coordinated metabolic signature compatible with alterations in mitochondrial metabolism, nitric oxide biosynthesis, one‐carbon metabolism, membrane remodelling and oxidative stress. These findings identify biological pathways that warrant further mechanistic investigation and may inform the development of future therapeutic strategies. Plasma creatinine and ADMA emerge as promising, inexpensive and readily accessible candidate biomarkers of disease severity that warrant validation in larger and clinically more heterogeneous FSHD cohorts.

## Funding

This study was supported by the French FSHD Society (Amis FSH grant to MS). MS is supported by an INSERM clinician‐scientist position (CIHU INSERM) (Institut National de la Santé et de la Recherche Médicale).

## Ethics Statement

This study was conducted in accordance with the 1964 Declaration of Helsinki and its later amendments. Plasma samples were collected after the participants (patients and controls) provided written informed consent for the use of samples from existing biological sample collections. This biological collection was approved by the Ethical Committee of the University Hospital of Angers (Comité de Protection des Personnes CPP Ouest II, Angers, France; Identification Number CPP CB 2014/02; Declaration Number DC‐2011‐1467; Authorization Number AC‐2023‐5473). The associated data collection was authorized by the local ethics committee under Reference 2023‐156.

## Conflicts of Interest

The authors declare no conflicts of interest.

## Supporting information


**Figure S1:** Lack of inter‐centre batch effects. PCA scatter plots illustrating the distribution of samples according to the collection city. Blue dots refer to Angers (*n* = 50), orange to Modena (*n* = 40), and green to Naples (*n* = 28). Ellipses represent the 95% confidence intervals. The overlapping ellipses indicate that no clear differences between cities are observed in the PCA.
**Figure S2:** PCA analysis of control and FSHD samples. Scatter plots illustrating the distribution of FSHD (purple, *n* = 55) and control (green, *n* = 63) samples, based on principal component analysis (PCA). The red ellipse represents Hotelling's T^2^ 99% confidence limit, highlighting one outlier that was subsequently removed from the study. The purple and green ellipses represent the 95% confidence intervals for the FSHD and control groups, which are overlapping, showing no clear distinction between the groups.
**Figure S3:** Metabolic pathway enrichment analysis. The analysis was performed using a predefined literature‐derived panel of 30 mitochondria‐associated metabolites indicated in Table S3. Match Status indicates the number of dataset metabolites mapped to the pathway relative to the total number of compounds it contains. The *p*‐value was calculated using the Global Test, which evaluates the association between a pathway's metabolite profile and disease status. FDR corresponds to the Benjamini–Hochberg‐adjusted *p*‐value, correcting for multiple comparisons across pathways. Impact reflects the relative importance of the matched metabolites within each pathway's topological structure, based on relative‐betweenness centrality. Pathways with FDR < 0.05 were considered significantly enriched.
**Table S1:** List of metabolites contributing the most to the OPLS‐DA model.
**Table S2:** Top features identified for each machine‐learning model with their corresponding AUC values.
**Table S3:** Literature‐derived panel of metabolites associated with mitochondrial metabolism or mitochondrial dysfunction used for mitochondrial pathway enrichment and machine‐learning ROC curve analyses. Metabolites quantified in our dataset were selected a priori from the literature based on reported associations with mitochondrial dysfunction and/or altered circulating concentrations in primary mitochondrial diseases. Reference numbers indicate the studies supporting the inclusion of each metabolite. KEGG identifiers are provided where available. C6 (C4:1‐DC) was excluded from the mitochondrial pathway enrichment analysis because no corresponding KEGG identifier is currently available.
**Table S4:** Key circulating metabolic alterations identified in FSHD1 patients and their biological relevance.

Supporting Information

## Data Availability

Individual‐level metabolomics data are included in the Supporting Information. Other data supporting the findings of this study are available from the corresponding author upon reasonable request.
