# Supplementary material for: Metabolomics Reveals Multiple Metabolic Alterations and Biomarkers of Disease Severity in Facioscapulohumeral Muscle Dystrophy
Source: J Cachexia Sarcopenia Muscle. 2026 Nov 6;17(6):e70414. doi: 10.1002/jcsm.70414 (PMC13743862; doi:10.1002/jcsm.70414)
Supplement: Supplementary file 1 [file JCSM-17-e70414-s001.pdf]

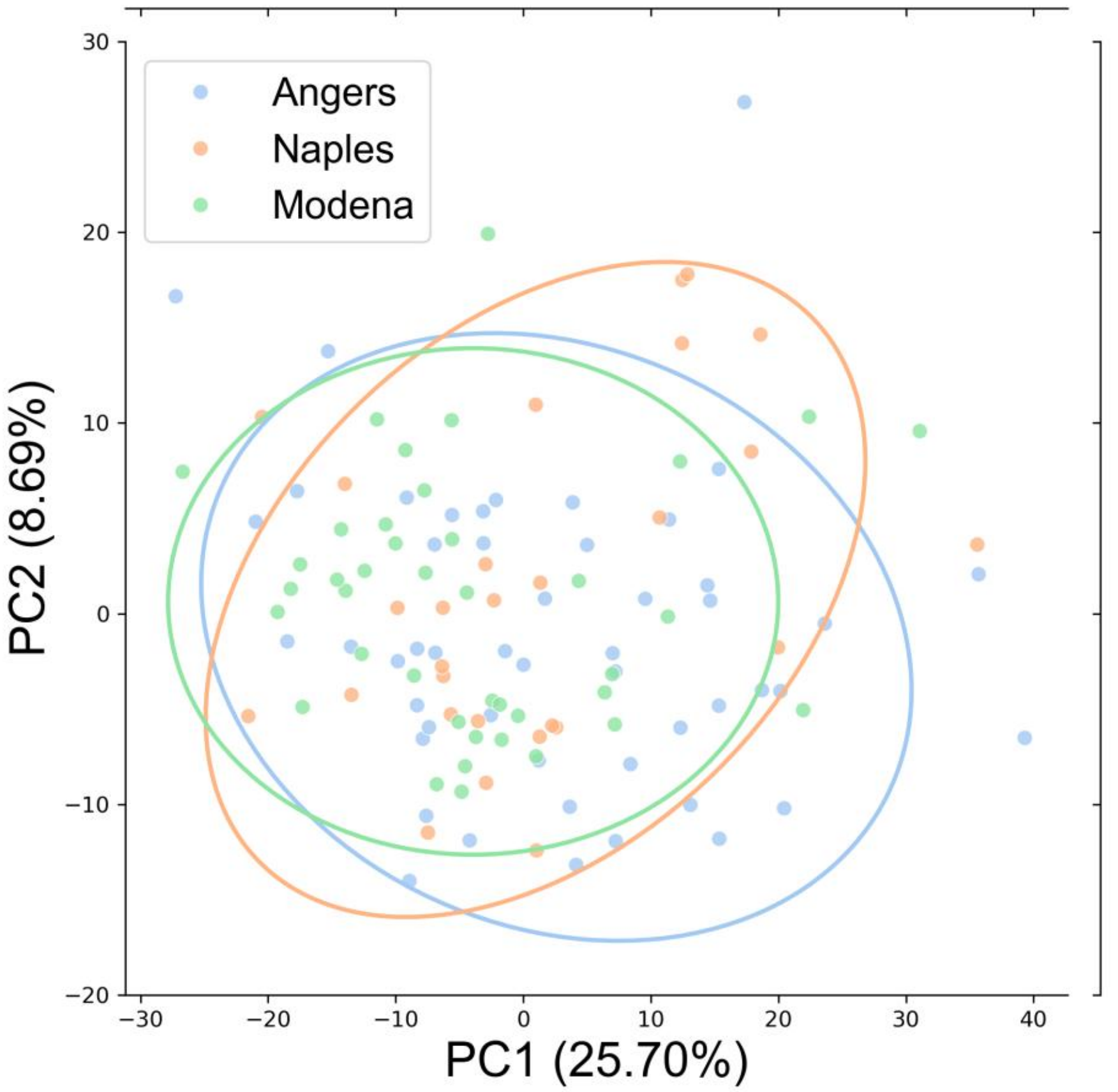

Supplementary Figure 1. Lack of inter-center batch effects. PCA scatter plots illustrating the distribution of samples according to the collection city, Blue dot refer to Angers (n=50), orange to Modena (n=40), and green to Naples (n=28). Ellipses represent the 95% confidence intervals. The overlapping ellipses indicate that no clear differences between cities are observed in the PCA.

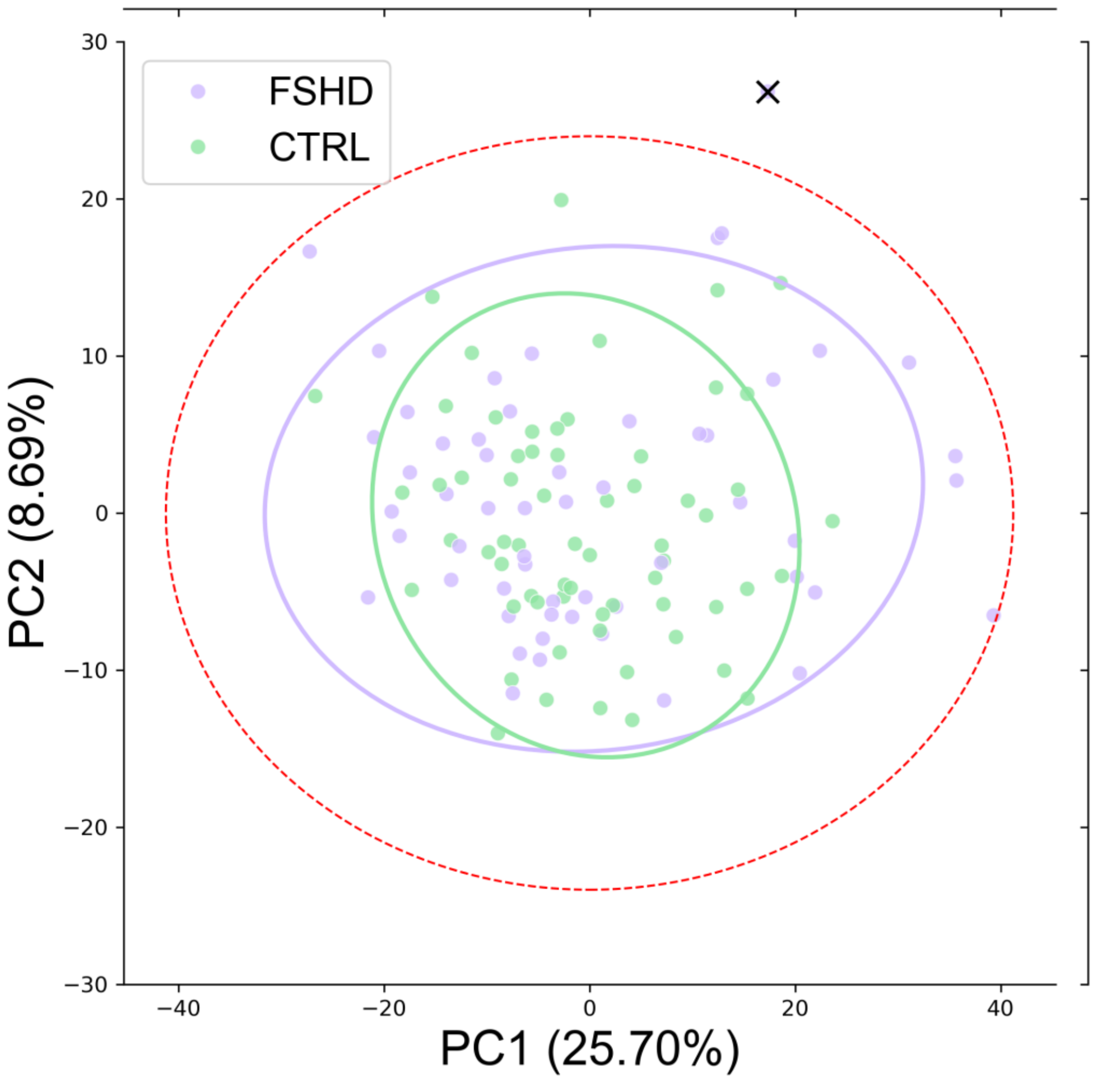

Supplementary Figure 2. PCA analysis of Control and FSHD samples. Scatter plots illustrating the distribution of FSHD (purple, n=55) and control (green, n=63) samples, based on Principal Component Analysis (PCA). The red ellipse represents Hotelling's T^2 99% confidence limit, highlighting one outlier that was subsequently removed from the study. The purple and green ellipses represent the 95% confidence intervals for the FSHD and Control groups which are overlapping, showing no clear distinction between group.

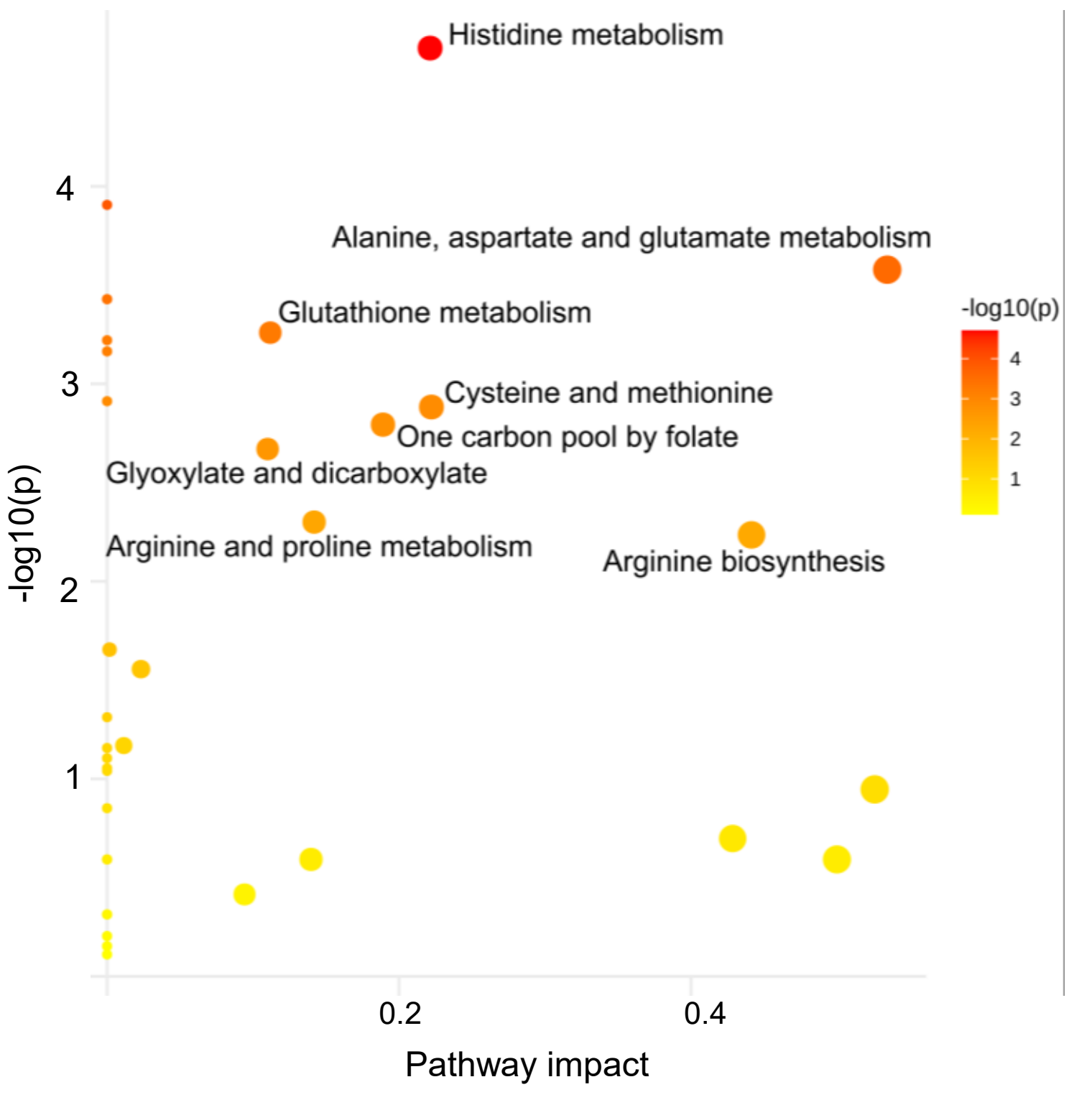

Pathway Name	Match Status	p-value	FDR	Impact
**Histidine metabolism**	3/16	2.0×10^{-5}	7.0×10^{-4}	0.221
**Beta-alanine metabolism**	2/21	1.2×10^{-4}	0.0022	0
**Alanine, aspartate and glutamate metabolism**	5/28	2.6×10^{-4}	0.0026	0.534
**Glycolysis or gluconeogenesis**	1/26	3.7×10^{-4}	0.0026	0
**Pyruvate metabolism**	1/23	3.7×10^{-4}	0.0026	0
**Glutathione metabolism**	3/28	5.5×10^{-4}	0.0030	0.111
**Porphyrin metabolism**	2/31	6.0×10^{-4}	0.0030	0
**Nitrogen metabolism**	2/6	6.8×10^{-4}	0.0030	0
**Butanoate metabolism**	1/15	0.0012	0.0046	0
**Cysteine and methionine metabolism**	3/33	0.0013	0.0046	0.222
**One carbon pool by folate**	5/26	0.0016	0.0051	0.189
**Glyoxylate and dicarboxylate metabolism**	4/32	0.0021	0.0062	0.110
**Arginine and proline metabolism**	3/36	0.0050	0.0135	0.141
**Arginine biosynthesis**	5/14	0.0058	0.0145	0.441

Supplementary Figure 3. Metabolic pathway enrichment analysis. The analysis was performed using a predefined literature-derived panel of 30 mitochondrial-associated metabolites indicated in Supplementary Table 3. Match Status indicates the number of dataset metabolites mapped to the pathway relative to the total number of compounds it contains. The p-value was calculated using the Global Test, which evaluates the association between a pathway's metabolite profile and disease status. FDR corresponds to the Benjamini-Hochberg-adjusted p-value, correcting for multiple comparisons across pathways. Impact reflects the relative importance of the matched metabolites within each pathway's topological structure, based on relative-betweenness centrality. pathways with FDR < 0.05 were considered significantly enriched.

Supplementary Table 1 List of metabolites contributing the most to the OPLS-DA model

Metabolite	VIP	p(corr)
Creatinine	1.951	-0.583
ADMA	1.752	0.519
Histidine	1.674	-0.502
Choline	1.562	0.461
Cortisone	1.469	-0.429
Asparagine	1.450	-0.402
Methionine	1.336	-0.397
Lactate	1.313	0.368
SM 34:2	1.308	0.362
Cortisol	1.297	-0.373
LPC 17:0	1.297	-0.350
PI 16:0_20:4	1.258	0.302
TG 16:0_36:2	1.255	0.244
PE 40:4	1.221	0.274
PC O-38:0	1.217	-0.282
Cer d18:2/22:0	1.205	0.327
Glutamate	1.176	0.307
Cer d18:2/24:0	1.174	0.283
TG 18:1_36:6	1.165	-0.274
Hex-Cer d18:2/22:0	1.152	0.334
TG 18:1_33:3	1.136	-0.242
TG 14:0_34:3	1.114	-0.113
TG 18:2_32:0	1.114	0.115
Hex-Cer d18:2/24:0	1.111	0.312
TG 16:0_36:3	1.096	0.176
TG 16:0_36:6	1.094	-0.202
PG 16:0_18:1	1.081	0.317
TG 20:3_32:0	1.080	0.125
TG 18:2_34:1	1.080	0.158
Alanine	1.072	-0.295
TG 22:5_32:1	1.071	-0.119
PI 15:0_16:0	1.070	0.197
TG 16:1_32:2	1.051	-0.057
TG 17:0_34:3	1.043	-0.097
TG 20:4_34:2	1.038	0.118
PE 36:4	1.032	0.228
Ornithine	1.028	0.295
PC 34:4	1.026	-0.169
Cer d18:1/24:1	1.025	0.212
Hex2Cer d18:1/22:0	1.021	0.240
PC O-34:3	1.018	-0.221

Metabolites with VIP > 1 are those that contribute the most to the OPLS-DA model. The standardized loading value, p(corr), represents the correlation between each metabolite and the predictive component of the OPLS-DA model. A negative p(corr) indicates a decrease of the metabolite concentration in FSHD compared to controls, while a positive p(corr) reflects an increase metabolite concentration in FSHD within the OPLS-DA model. VIP: Variable Importance in the Projection. ADMA: asymmetric dimethylarginine; SM: sphingomyelin; LPC: lysophosphatidylcholine; PI: phosphatidylinositol; TG: triglyceride; PE: phosphatidylethanolamine; PC: phosphatidylcholine; Cer: ceramide; Hex-Cer: monohexosylceramide; PG: phosphatidylglycerol; Hex2Cer: dihexosylceramide.

Supplementary Table 2 Top features identified for each machine learning model with their corresponding AUC values

Lasso	Creatinine | ADMA | His | PG 16:0_18:1 | PI 18:1_18:2 | Cer d18:2/22:0 | PA 18:1_18:2 | Hex2Cer d18:1/24:0 | Cortisol | LPE 18:3 | PG 15:0_18:1 | DG 18:1_20:0 | PC O-38:0 | Gly | Choline | TG 22:5_32:1 | GCA | LPC 17:0 | 3-Met-His | Cer d18:1/25:0	0.827 ± 0.08
XGBoost	Creatinine | ADMA | His | PC 34:3 | TG 22:5_32:1 | TG 22:5_34:3 | Asn | CE 15:0 | Cortisol | PC O-38:0 | PI 18:1_18:2 | Cer d18:1/24:1 | Lac | PC 40:5 | PI 16:0_17:2 | PS 38:4 | TG 20:0_32:3 | SPBP d18:1 | PC O-36:2 | Cer d18:2/23:0	0.805 ± 0.08
Random Forest	Creatinine | ADMA | Asn | His | Lac | Cortisol | PC 36:6 | Cortisone | TG 22:5_34:3 | Choline | TG 22:5_32:1 | PA 16:2_18:1 | TG 18:1_33:3 | PC O-38:0 | Cer d18:2/22:0 | Met | PI 16:0_17:2 | Gln | Cer d18:1/25:0 | PI 16:0_20:4	0.770 ± 0.09
Ridge	Creatinine | ADMA | PA 18:1_18:2| His | PG 16:0_18:1 | Choline | PG 15:0_18:1 | Met | Lac | SDMA | PI 18:1_18:2 | PI 16:0_22:1 | Cortisol | Cer d18:1/25:0 | Cer d18:2/22:0 | Trigonelline | DG 18:1_20:0 | GABA | Sarcosine | Asn	0.769 ± 0.09
SVM	Creatinine | ADMA | PA 18:1_18:2 | His | PG 16:0_18:1 | Choline | Lac | Met | PG 15:0_18:1 | PI 18:1_18:2 | Cer d18:1/25:0 | PI 16:0_22:1 | SDMA | Cortisol | DG 18:1_20:0 | Trigonelline | Glu | Asn | Cer d18:2/22:0 | CA	0.737 ± 0.09

The 20 most important features for each model were determined based on SHAP value computations. Variables highlighted in italics represent those considered most important in the final analysis (Fig. 3). ADMA : asymmetric dimethylarginine; Asn : Asparagine; AUC : area under curve; CA : cholic acid; CE : cholesteryl esters; Cer : ceramides; DG : diacylglycerol; GABA : gamma aminobutyric acid; GCA : glycocholic acid; Gln : glutamine; Glu : glutamic acid; Gly : glycine; Hex2Cer : dihexosylceramide; His : histidine; Lac : lactate; LPC : lysophosphatidylcholine; LPE : lysophosphatidylethanolamine; Met : methionine; PA : palmitic acid; PC : phosphatidylcholine; PG : phosphatidylglycerol; PI : phosphatidylinositol; PS : phosphatidylserine; SDMA : symmetric dimethyl arginine; SPBP : sphingosine phosphate; TG : triglyceride.

Supplementary Table 3 Literature-derived panel of metabolites associated with mitochondrial metabolism or mitochondrial dysfunction used for mitochondrial pathway enrichment and machine-learning ROC curve analyses. Metabolites quantified in our dataset were selected *a priori* from the literature based on reported associations with mitochondrial dysfunction and/or altered circulating concentrations in primary mitochondrial diseases. Reference numbers indicate the studies supporting the inclusion of each metabolite. KEGG identifiers are provided where available. C6 (C4:1-DC) was excluded from the mitochondrial pathway enrichment analysis because no corresponding KEGG identifier is currently available.

Biocrates Name	Name	KEGG ID	References
Lac	Lactate	C00186	1-4
Ala	Alanine	C00041	1-4
Arg	Arginine	C00062	1-3,5
Asn	Asparagine	C00152	1,3-4
Asp	Aspartic acid	C00049	3
Cys	Cysteine	C00097	1-2
Cit	Citrulline	C00327	1,5
Gln	Glutamine	C00064	1-2
Glu	Glutamate	C00025	1,3
Gly	Glycine	C00037	1,2
His	Histidine	C00135	1,3
Ile	Isoleucine	C00407	1,2,5
Leu	Leucine	C00123	1,2,5
Lys	Lysine	C00047	1,3
Met	Methionine	C00073	1
Pro	Proline	C00148	1,2
Ser	Serine	C00065	1,3
Taurine	Taurine	C00245	1,3
Thr	Threonine	C00188	1,2
Tyr	Tyrosine	C00082	2
Val	Valine	C00183	1,2,5
ADMA	Asymmetric dimethylarginine	C03626	5
Betaine	Betaine	C00719	3
Kynurenine	Kynurenine	C00328	3,4
GCA	Glycocholic acid	C01921	3,4
Xanthine	Xanthine	C00385	3,6
C0	Carnitine	C00487	7
C2	Acetylcarnitine	C02571	4
C3	Propionylcarnitine	C03017	4
C6 (C4:1-DC)	Hexanoylcarnitine	-	4

References

[1] Shayota BJ. Biomarkers of mitochondrial disorders. Neurotherapeutics. 2024 Jan;21(1):e00325. doi: 10.1016/j.neurot.2024.e00325
[2] Hubens WHG et al. Blood biomarkers for assessment of mitochondrial dysfunction: Anexpert review. Mitochondrion. 2022 Jan;62:187-204. doi: 10.1016/j.mito.2021.10.008.
[3] Buzkova J et al. Metabolomes of mitochondrial diseases and inclusion body myositis patients: treatment targets and biomarkers. EMBO Mol Med. 2018 Dec;10(12):e9091. doi: 10.15252/emmm.201809091.
[4] Thompson Legault et al. A Metabolic Signature of Mitochondrial Dysfunction Revealed through a Monogenic Form of Leigh Syndrome. Cell Rep. 2015 Nov 3;13(5):981-9. doi: 10.1016/j.celrep.2015.09.054.
[5] El Hattab A et al. Restoration of impaired nitric oxide production in MELAS syndrome with citrulline and arginine supplementation. Mol Genet Metab. 2012 Apr;105(4):607-14. doi: 10.1016/j.ymgme.2012.01.016
[6] Bocca et al. A Plasma Metabolomic Signature Involving Purine Metabolism in Human Optic Atrophy 1 (OPA1)-Related Disorders. Invest Ophthalmol Vis Sci. 2018 Jan 1;59(1):185-195. doi: 10.1167/iovs.17-23027.
[7]Alban C et al. The Relationship between Mitochondrial Respiratory Chain Activities in Muscle and Metabolites in Plasma and Urine: A Retrospective Study. J Clin Med 2017;6:31. doi: 10.3390/jcm6030031.

Supplementary Table 4. Key circulating metabolic alterations identified in FSHD1 patients and their biological relevance

Marker	Change	Pathway / biological process	Reported relevance in muscle / neuromuscular disease	Interpretation in the present study
**Creatinine**	↓	Creatine metabolism; muscle mass	Surrogate marker of muscle mass; decreased in sarcopenia, Duchenne muscle dystrophy and spinal muscle dystrophy	Strong effect size and correlation with clinically relevant functional outcomes
**ADMA**	↑	Nitric oxide (NO) metabolism<br><br>One carbon metabolism<br><br>Oxidative stress	Endogenous inhibitor of NO synthase; implicated in metabolic dysregulation and muscle perfusion; insulin resistance, critical illness, ALS, MELAS, ageing	Suggests alteration of NO-related pathways and one carbon metabolism
**Histidine**	↓	Amino acid metabolism<br><br>One carbon metabolism	Reduced levels reported in conditions associated with muscle wasting and altered protein turnover; previously reported in one FSHD study	Consistent with increased muscle catabolism and altered one carbon metabolism
**Asparagine**	↓	Amino acid metabolism; mitochondrial-linked pathways	Altered in metabolic and mitochondrial disorders	Supports systemic metabolic imbalance compatible with mitochondrial involvement
**Lactate**	↑	Increased glycolysis in response to mitochondrial dysfunction	Established marker of impaired oxidative metabolism/mitochondrial dysfunction	Consistent with mild mitochondrial dysfunction
**GDF-15**	↑	Stress response; mitochondrial signaling	Pleiotropic biomarker, best currently available maker of mitochondrial dysfunction; increased levels reported in inclusion body myositis and Charcot Marie Tooth patients	Consistent with subtle mitochondrial stress

Supplementary multivariate and machine learning methods

Multivariate analyses were performed including Principal Component Analysis (PCA) for unsupervised data exploration and outlier detection, and Orthogonal Partial Least Squares Discriminant Analysis (OPLS-DA) for supervised classification using Simca-P + v17.0 (Umetrics, Umea, Sweden). Allowing model optimization through iterative variable exclusion. The selection of metabolites of interest was guided by multiple complementary outputs, including the S-plot, the loading column plot with jackknife confidence intervals, the coefficient plot, and the variable importance in projection (VIP) plot. This combined approach aimed to reduce the risk of overfitting and minimize prediction variability. Only metabolites with a VIP score greater than 1 were considered relevant contributors to the metabolomic footprint. Models with cumulative predictive abilities Q^2cum (prediction goodness) > 0.5 were deemed discriminative. Model robustness was evaluated via CV-ANOVA (cross-validation analysis of variance), R2Ycum (fit goodness) values, and a permutation test (assessing overfitting risk) using 7-fold cross validation. Additionally, p(corr) values (correlation-scaled loadings) were examined to identify variables most consistently correlated with class discrimination.

A machine learning pipeline was also implemented to benchmark classification performance and enhance feature identification. Five algorithms were tested: logistic regression with Ridge and Lasso penalization, XGBoost, Random Forest and Support Vector Machine (SVM). A repeated nested cross-validation scheme was used, [13] with an outer 15-fold loop for model evaluation and an inner 5-fold loop for hyperparameter tuning. Grid search was used for hyperparameter optimization, and the best model was selected based on validation performance. Final models were trained on the full outer training set and evaluated on the outer test fold. This procedure was repeated thirty times with different random splits, and the mean area under the ROC curve was calculated across all 150 test folds enabling comparison of model performance. To interpret model predictions and assess feature contributions, SHAP values were computed for each selected metabolite, providing a transparent and theoretically grounded measure of feature importance.

All data processing was conducted using SIMCA (17.0), and Python (3.9) with the packages: NumPy (1.26.3), Pandas (2.1.4) SciPy (1.11.4), scikit-learn (1.3.0), xgboost (1.7.3), SHAP (0.41.0), seaborn (0.12.2), and matplotlib (3.8.0).

Metabolic pathway analysis

Pathway analysis was performed using MetaboAnalyst 6.0[1] (www.metaboanalyst.ca) with the Homo sapiens KEGG pathway library (December 2024 release). A predefined panel of 30 metabolites (see Supplementary Table 3) was manually curated from those reliably quantified in our dataset based on published evidence of their direct or indirect involvement in mitochondrial metabolism, irrespective of their statistical significance in the present study, and successfully mapped to KEGG identifiers (n=29). Pathway enrichment analysis was performed using the Global Test algorithm, which evaluates the association between the metabolite profile of each pathway and disease status (FSHD versus controls). Pathway topology analysis was based on relative-betweenness centrality, and all compounds included in the selected KEGG library were used as the reference metabolome. P-values were adjusted for multiple testing using the Benjamini–Hochberg false discovery rate (FDR), and pathways with an FDR < 0.05 were considered statistically significant.

[1]Pang, Z., Lu, Y., Zhou, G., Hui, F., Xu, L., Viau, C., Spigelman, A., MacDonald, P., Wishart, D., Li, S., and Xia, J. (2024) MetaboAnalyst 6.0: towards a unified platform for metabolomics data processing, analysis and interpretation Nucleic Acids Research Nucleic Acids Res. 2024 Jul 5;52(W1):W398-W406. doi: 10.1093/nar/gkae253.
